# Effect of Dairy Matrix on the Postprandial Blood Metabolome

**DOI:** 10.3390/nu13124280

**Published:** 2021-11-27

**Authors:** Rebekka Thøgersen, Kristian Leth Egsgaard, Louise Kjølbæk, Klaus Juhl Jensen, Arne Astrup, Marianne Hammershøj, Anne Raben, Hanne Christine Bertram

**Affiliations:** 1 Department of Food Science, Aarhus University, Agro Food Park 48, DK-8200 Aarhus, Denmark; rebekka.thoegersen@food.au.dk (R.T.); 201508816@post.au.dk (K.L.E.); marianne.hammershoj@food.au.dk (M.H.); 2 Department of Nutrition, Exercise and Sports, University of Copenhagen, DK-1958 Frederiksberg, Denmark; louisekjoelbaek@nexs.ku.dk (L.K.); ara@novo.dk (A.A.); ara@nexs.ku.dk (A.R.); 3 Arla Foods Amba, DK-8200 Aarhus, Denmark; kljes@arlafoods.com; 4 Healthy Weight Center, Novo Nordisk Foundation, DK-2900 Hellerup, Denmark; 5 Clinical Research, Copenhagen University Hospital—Steno Diabetes Center Copenhagen, DK-2730 Herlev, Denmark

**Keywords:** amino acid absorption, food structure, glucono-δ-lactone, NMR metabolomics, cheese

## Abstract

This study investigated the postprandial plasma metabolome following consumption of four dairy matrices different in texture and structure: cheddar cheese (Cheese), homogenized cheddar cheese (Hom. Cheese), and micellar casein isolate (MCI) with cream (MCI Drink) or a MCI Gel. An acute, randomized, crossover trial in male participants (n = 25) with four test days was conducted. Blood samples were collected during an 8-h postprandial period after consumption of a meal similar in micro- and macronutrients containing one of the four dairy matrices, and the metabolome was analyzed using nuclear magnetic resonance (NMR) spectroscopy. A liquid dairy matrix (MCI Drink) resulted in a faster absorption of amino acids compared to products, representing either a semi-solid (MCI Gel and Hom. Cheese) or solid (Cheese) dairy matrix. For the MCI Gel, plasma concentration of acetic acid and formic acid increased approximately 2 h following consumption, while 3-hydroxybyturate and acetoacetic acid increased approximately 6 h after consumption. The structure and texture of the dairy matrix affected the postprandial absorption of amino acids, as revealed by the plasma metabolome. Our study furthermore pointed at endogenous effects associated with consumption of dairy products containing glucono-δ-lactone.

## 1. Introduction

Dairy products can contain high amounts of saturated fats, which, traditionally, have been associated with increased risk of cardiovascular disease (CVD) [1]; however, studies have indicated that a potentially harmful impact of saturated fatty acids on plasma lipids depends on the type of dairy product consumed, and the association between dairy product consumption may even be neutral or protective [2,3,4]. 

The complexity between type of dairy product consumed and impact on CVD indicates an effect of the food matrix. Evidence is increasing that the nutritional properties of a food is not only determined by its single nutrients, but also by the complex food structure in which the nutrients are embedded [5,6]. Thus, studies indicate that the food matrix can affect digestion and absorption of the nutrients in a given food [2,7]. In particular, the dairy matrix, comprising both solid matrices (cheese), semi-solid matrices (yoghurt, crème fraiche), and liquids (milk, cream), receives attention [8]. In vitro and in vivo studies have indicated that the physical structure and processing of dairy products may affect bioavailability of nutrients [8]. In vitro studies have shown that the texture and composition of cheeses affect their disintegration upon digestion [9,10]. A low initial hardness, cohesiveness, and chewiness of the cheeses was associated with a higher disintegration at the end of gastric digestion [9]. Lamothe et al. (2017) investigated the release of nutrients in a simulated gastrointestinal environment from three structurally different dairy matrices: milk, yoghurt, and cheese [11]. The results showed that the cheeses were more resistant to protein and lipid digestion than milk and yoghurt. Barbé et al. (2013) investigated protein digestion and amino acid (AA) absorption in six mini pigs consuming four different dairy matrices: skimmed milk and rennet gels, either heated or unheated [12]. They found that heat treatment increased the retention time in the stomach and that gelation delayed gastric emptying. Further, analysis of postprandial plasma samples showed that gelation resulted in delayed and lower AA absorption in the postprandial phase [12]. A recent human study investigated the postprandial blood AA concentration after consumption of different dairy matrices [13]. Here, a higher postprandial blood concentration of essential AA (EAA) was observed after consumption of yoghurt compared to milk and cheese. In addition, higher fat content resulted in a slower protein digestion [13]. Thus, several studies have indicated that the nature of the dairy matrix affects digestion and nutrient absorption; however, human studies investigating the impact of the dairy matrix on AA absorption and postprandial responses are sparse. Therefore, during an acute, randomized, crossover human trial, the present study investigated the effect of consuming four different dairy matrices on blood plasma metabolites during an 8-h postprandial period using 1H nuclear magnetic resonance (1H NMR) spectroscopy. The four experimental dairy matrices, a cheddar cheese (Cheese), a homogenized cheddar cheese (Hom. Cheese), micellar casein isolate with cream (MCI Drink), or a gel made from the MCI Drink (MCI Gel), differed in texture and structure and were served as meals in which the nutrient compositions were similar. This study is part of the interdisciplinary DAIRYMAT project. Detailed description of the study design and primary outcomes [14], a thorough characterization of the experimental dairy products [15], and the effect of the experimental diets on postprandial phospholipids [16] have previously been published.

## 2. Materials and Methods

### 2.1. Study Design and Participants

A total of 25 men aged 19–40 years with a body mass index (BMI) between 20.0 and 24.5 kg/m^2^ were enrolled for this acute, randomized, crossover trial, previously described in detail in Kjølbæk et al., 2021 [14]. The study was conducted from September 2018 to March 2019 at the Department of Nutrition, Exercise, and Sports, University of Copenhagen, Denmark, and consisted of four test days. Use, or use within the previous 3 months, of prescription medication, except non-prescription primary analgesics (non-steroidal anti-inflammatory drugs and paracetamol) and glucocorticoids for topical use, was not allowed. A total of 21 participants completed a minimum of two test days and were included in the analyses, and 18 participants completed all four test days. The participants consumed a standardized meal before 22:00 in the evening prior to the test days. In addition, alcohol, medication, and moderate-to-vigorous physical activities were to be avoided 48 h prior to the test days. Upon arrival at each test day, the participants were weighed and fasting blood samples were collected. Subsequently, one of four iso-energetic test meals and 1.5 g paracetamol was consumed over 15 min. The test meal consisted of one of the dairy matrices, bread and water. During the following 8 h, blood samples were collected at 30, 60, 90, 120, 180, 240, 300, 360, 420, and 480 min. In order to obtain plasma, blood samples were transferred to heparin tubes and centrifuged at 2500 g for 10 min at 4 °C. Plasma samples were stored at −80 °C until analysis. The study was registered at clinicaltrial.gov (NCT03656367) (accessed on 4 September, 2018).

### 2.2. Test Meals

At each test day, the participants consumed one of four dairy matrices served with bread and water [14]. The four dairy matrices were (I) a commercial full-fat cheddar cheese (Cheese) (Arla Foods Ltd., Leeds, UK), (II) a homogenized cheddar cheese (Hom. Cheese), (III) a micellar casein isolate (MCI) with cream and salt (MCI Drink), and (IV) a gel made from the MCI Drink by glucono-δ-lactone addition (GDL) (MCI Gel), as described by Schmidt et al. (2020) [15]. The meals were similar in micro and macronutrient composition, but the dairy matrices differed in texture and structure. Since the dairy matrices differed in carbohydrate and salt content, lactose, potato starch, and salt were added to the bread to adjust for these differences to obtain meals that contained 54 g protein, 72 g carbohydrate, 68 g of fat, and 1.53–1.57 g sodium chloride.

### 2.3. NMR Spectroscopy

Plasma samples were thawed, and 350 µL plasma was mixed with 350 µL phosphate buffer in D_2_O (pH 7.4). A volume of 600 µL was subsequently transferred to a 5-mm NMR tube. NMR spectroscopy was conducted using a Bruker Avance IVDr 600 MHz spectrometer, operating at a frequency of 600.13 MHz (Bruker BioSpin, Rheinstetten, Germany). The NMR spectrometer was equipped with a 5 mm 1H TXI probe and a Bruker SampleJet set to 4 °C. An in vitro diagnostics research (IVDr) tool (Avance IVDr, IVDr methods, User manual, version 3, Bruker Corporation, Schwitzerland) was used to analyze the samples. Four experiments were applied in automation for each sample: 1D Nuclear Overhauser Effect Spectroscopy (NOESY), 2D J-resolved experiment (JRES), Carr−Purcell− Meiboom−Gill (CPMG), and difference spectroscopy (DIFF). The CPMG spectra were used for metabolite quantification using the following parameters: dummy scans: 4; number of scans: 32; relaxation delay: 4 s; acquisition time: 3.10 s; spectral width: 19.84 ppm. Plasma metabolites were automatically quantified by B.I.Quant-PS (version 2.0, Bruker BioSpin, Rheinstetten, Germany). In total, 41 plasma metabolites were detected and quantified. The quality of the quantification for the individual metabolites was evaluated, and as a result, 22 metabolites were included in the data analysis. Detection of 22 metabolites in human plasma using the Bruker IVDR platform is consistent with a former ring trial, where quantification of 24 metabolites were reported [17]. Data on analytical variance and reproducibility for the applied NMR IVDr SOP obtained can be found elsewhere [17].

### 2.4. Statistics

Data were analyzed using mixed effect models to analyze the effects of meal, time, and meal-time interactions. Meal and time were treated as fixed factors and the participant as the random factor. False discovery rate (FDR) adjustment using the Benjamini and Hochberg approach [18] was conducted in order to control for multiple testing. FDR-adjusted *p*-values are denominated q-values. If significant meal-time interactions were found (q-value < 0.05), Tukey’s multiple comparison test was used to identify time points with significant differences between meals. *p*-values < 0.05 were considered significant. Data is shown as mean ± SEM. GraphPad Prism (Version 9) was used for statistical analyses and graphs.

## 3. Results

A total of 21 participants completed a minimum of two test days and were included in the analyses, and 18 participants completed all four test days. Due to the lack of sufficient sample material, a total of 851 plasma samples were analyzed using NMR spectroscopy, and data analysis was performed on a total of 22 plasma metabolites. For all of the analyzed metabolites, except formic acid, a significant effect of time was found (q < 0.05).

### 3.1. Amino Acid Concentrations in Plasma

**Figure 1:**
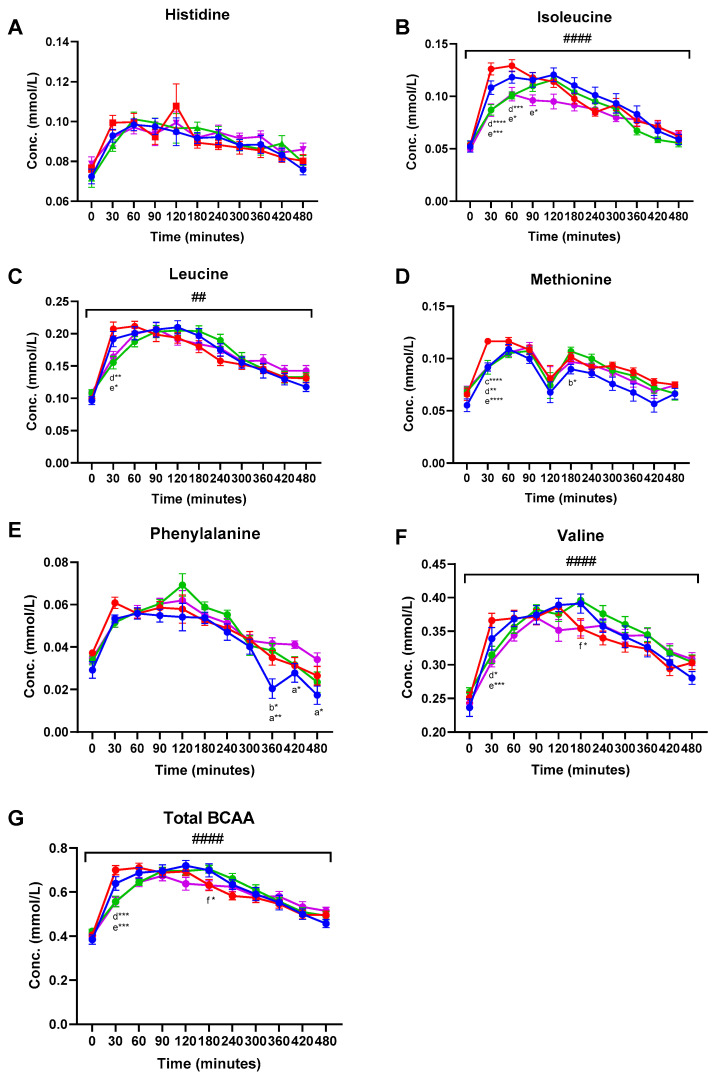
Postprandial plasma concentrations (mean ± SEM) of essential amino acids: (**A**) Histidine, (**B**) isoleucine, (**C**) leucine, (**D**) methionine, (**E**) phenylalanine, (**F**) valine, and (**G**) total branched chain amino acids. ● MCI Drink, ● MCI Gel, ● Hom. Cheese, ● Cheese. # indicate significant meal-time interactions: # (q < 0.05), ## (q < 0.005), ### (q < 0.0005) #### (q < 0.0001). Letters indicate significant differences in post-hoc tests: ^a^ MCI gel vs. Cheese, ^b^ MCI gel vs. Hom. Cheese, ^c^ MCI gel vs. MCI drink, ^d^ MCI drink vs. Hom. Cheese, ^e^ MCI drink vs. Cheese, ^f^ Hom. Cheese vs. Cheese. Asterisks indicate level of significance: * (*p* < 0.05), ** (*p* < 0.005), *** (*p* < 0.0005) **** (*p* < 0.0001).

Amino acids (AA) detected and included in the data analysis were alanine, glutamine, glycine, histidine, leucine, isoleucine, methionine, phenylalanine, tyrosine, and valine (Supplementary Material, Table S1). Plasma concentrations of EAA and total branched chain AA (BCAA) are shown in Figure 1. Significant meal-time interactions were found for alanine (q < 0.001), leucine (q = 0.001), isoleucine (q < 0.001), tyrosine (q < 0.001), valine (q < 0.001), and total BCAA (q < 0.001). A near-significant meal-time interaction was found for phenylalanine (q = 0.05). Significant meal effects were observed for isoleucine (q = 0.041), methionine (q = 0.041), and phenylalanine (q = 0.041) (Figure 1 and Table S1). Tukey’s multiple comparisons test was used to identify significant differences between treatment groups at specific time points during the postprandial period. In general, consumption of the MCI Drink resulted in the highest total plasma AA concentration 30 min post-consumption. Hence, significantly higher plasma concentration of leucine, isoleucine, methionine, tyrosine, valine, and total BCAA was found 30 min following consumption of the MCI Drink compared to Cheese and Hom. Cheese (Figure 1 and Table S1).

Among the remaining analyzed metabolites, significant meal effects were found for acetic acid (q < 0.001) and formic acid (q = 0.041). Significant meal-time interactions were found for acetic acid (q < 0.001), acetoacetic acid (q < 0.001), 3-hydroxybutyric acid (q = 0.037), and pyruvic acid (q < 0.001) (Table 1). From 120 min following consumption of the MCI Gel, an increasing plasma concentration of acetic acid was observed. From 180 to 420 min, significantly higher acetic acid concentrations in plasma were observed after consumption of MCI Gel compared to all of the other three products (Table 1). For formic acid, increased plasma concentrations were also observed from 180 min after MCI Gel consumption compared to the other products, although only reaching significance at 180 min compared to the MCI Drink, at 240 min compared to Hom. Cheese and Cheese, at 360 min compared to MCI Drink, at 420 min compared to MCI Drink and Hom. Cheese, and at 480 min compared to Hom. Cheese and Cheese (Table 1). 

### 3.2. Plasma Metabolites

**Figure 2:**
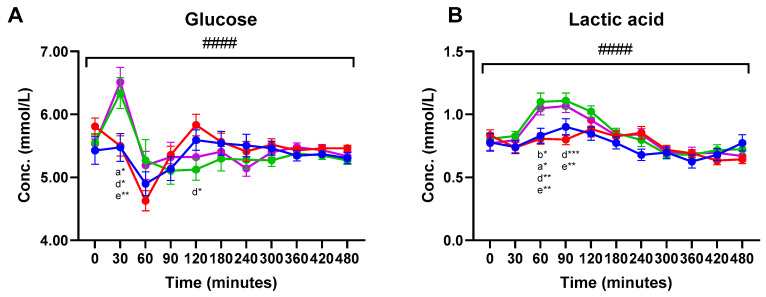
Postprandial plasma concentrations (mean ± SEM) of (**A**) glucose and (**B**) lactic acid. ● MCI Drink, ● MCI Gel, ● Hom. Cheese, ● Cheese. # indicate significant meal-time interactions: #### (q <0.0001). Letters indicate significant differences in post-hoc tests: ^a^ MCI gel vs. Cheese, ^b^ MCI gel vs. Hom. Cheese, ^c^ MCI gel vs. MCI drink, ^d^ MCI drink vs. Hom. Cheese, ^e^ MCI drink vs. Cheese, ^f^ Hom. Cheese vs. Cheese. Asterisks indicate level of significance: * (*p* <0.05), ** (*p* <0.005), *** (*p* <0.0005)). For 3-hydroxybutyric acid, a significantly higher plasma concentration was found at 360 min following consumption of the MCI Gel compared to the MCI Drink (Table 1). For acetoacetic acid, significant differences were found at 360 min. Thus, significantly higher plasma concentrations of acetoacetic acid were found after consumption of MCI Gel compared to the MCI Drink and Hom. Cheese, and a significantly higher concentration was found after Cheese consumption compared to the MCI Drink. For pyruvic acid, significant differences were found after 60 and 90 min. At 60 min, Cheese consumption resulted in significantly higher plasma pyruvic acid concentrations compared to MCI Gel and near-significant differences were higher compared to the MCI Drink (*p* = 0.069). After 90 min, significantly higher concentrations were found after consumption of Cheese and Hom. Cheese compared to the MCI Drink.

Significant meal-time interactions were found for postprandial plasma concentrations of glucose and lactic acid (q < 0.0001) (Figure 2). For both glucose and lactic acid, increased plasma concentrations were observed within 30 min after consumption of Cheese and Hom. Cheese compared to the two MCI products. Thus, Cheese consumption significantly increased glucose plasma concentrations at 30 min compared to the MCI Drink and MCI Gel. The Hom. Cheese significantly increased glucose concentrations at 30 min compared to the MCI Drink. A near-significant higher glucose concentration was observed 30 min after consumption of Hom. Cheese compared to the MCI Gel (*p* = 0.060). For lactic acid, Cheese and Hom. Cheese significantly increased plasma concentrations 60 min following consumption compared to the two MCI products. At 90 min, lactic acid concentrations were significantly increased after consumption of Cheese and Hom. Cheese compared to the MCI Drink (Figure 2).

## 4. Discussion

The present study investigated the effect of four different dairy matrices, different in texture and structure, and consumed within a meal similar in micro- and macronutrients, on the plasma metabolome. In general, the MCI Drink resulted in the highest plasma concentration of AAs 30 min after consumption, indicating a faster absorption of AAs after consumption of the liquid food matrix. Thus, significantly higher plasma concentrations of isoleucine, leucine, valine, methionine, tyrosine, and total BCAA were found 30 min following consumption of MCI Drink compared to Cheese and Hom. Cheese. When comparing the MCI Drink and MCI Gel, a significantly higher concentration after consumption of the MCI Drink was only reached for methionine after 30 min. Horstman et al. (2021) previously found that consumption of yoghurt resulted in a higher postprandial blood concentration of EAA compared to milk and cheese [13]. The influence of the dairy matrix on postprandial AA absorption might be associated with the accessibility of proteins to digestion enzymes, which has previously been correlated to protein digestion kinetics in cheese [11]. Previous in vitro studies found that disintegration upon digestion was affected by texture and composition of the dairy matrix [9,10,11]. For cheeses, disintegration of the food matrix is necessary in order for pepsin to be able to access the proteins, and Lamothe et al. (2017) found that, in contrast to milk and yoghurt, which were disintegrated from the beginning of the digestion, cheese was gradually disintegrated during digestion [11]. The faster AA absorption after the MCI Drink and MCI Gel consumption might also be related to the gastric emptying rate, since previous studies have indicated that dairy matrices with increased viscosity decrease the rate of gastric emptying [12,19,20]. The clinical impact of the observed differences in AA absorption rate among the dairy products remains to be further elucidated. Although AA availability, in particular BCAA, is expected to be of importance for postprandial muscle protein synthesis, the exact threshold in plasma concentration remains to be established. A recent study found no significant differences in milk and insect (lesser mealworm) protein sources’ potential to stimulate muscle protein synthesis, even though postprandial plasma concentration of leucine was markedly and significantly higher after ingestion of milk protein compared with insect protein [21]. Of other clinical effects, it could be speculated that higher postprandial blood concentration of BCAA could stimulate secretion of insulin; however, insulin analyses did not reveal a higher insulin level at 30–60 min postprandial for the MCT Drink [14].

Increased plasma concentrations of glucose and lactic acid were observed 30 min and 60–90 min following consumption of the Cheese and Hom. Cheese, respectively, compared to the MCI products. The increased lactic acid concentrations in plasma following consumption of the cheese products compared to the MCI products likely reflects a higher lactic acid content in the cheese products. Similarly, Cheese and Hom. Cheese increased pyruvic acid concentration 60–90 min following consumption. This increase might derive from pyruvic acid formed during fermentation and thus may be contained within the cheese products.

Since the cheese products did not contain lactose, lactose was added to the bread served with the products in order to adjust for these differences [14]. The lactose consumed through the bread might have been more accessible and more easily degraded compared to the lactose within the MCI products. Hence, this might account for the differences seen in the glucose response between the cheese products and the MCI products.

Intriguingly, from 120 min following consumption of the MCI Gel, a pronounced increase in plasma acetic acid was found. Similarly, but less pronounced, the concentration of formic acid increased from 180 min following consumption of the MCI Gel. The observed increase in these two carboxylic acids likely originates from endogenous formation following consumption of the MCI Gel. In contrast to the three other dairy matrices, GDL was added (3 g/100 g) in order to make the gelled product. GDL hydrolyses to form gluconic acid and lowers the pH [22]. Tsukahara et al. (2002) previously found that gluconic acid stimulated the bacterial formation of short-chain fatty acids, including acetate, in pig cecal digesta in vitro [23]. In addition, a previous study found that ingestion of gluconic acid increased the presence of fecal Bifidobacteria in humans [24]. Thus, the increased acetic acid concentrations in plasma might be caused by fermentation in the intestine following consumption of the MCI Gel. Formate can be formed as a byproduct from the production of acetate [25], which might explain the increase in formic acid concurrently with increased acetic acid concentrations. Later in the postprandial course (360 min), intake of the MCI Gel was associated with a significantly higher level of plasma 3-hydroxybutyrate and acetoacetic acid. The ketone bodies, 3-hydroxybutyrate and acetoacetic acid, are synthesized in the liver from short-chain fatty acids [26], and the observed increase in ketone bodies is thus most likely linked with increased serum acetate. This observation furthermore corresponds well with the in vitro digestion of the dairy matrices, where the MCI Gel resulted in the overall highest release of free fatty acids [15].

In conclusion, the present study demonstrated that intake of a liquid dairy matrix (MCI Drink) resulted in a faster absorption of AAs compared to products representing either a semi-solid (MCI Gel or Hom. Cheese) or solid (Cheese) dairy matrix. In addition, approximately 2 h following consumption of the MCI Gel, plasma concentration of acetic acid and formic acid increased. This might result from fermentation of gluconic acid originating from the GDL used to form the gelled product. Hence, the dairy matrix affected the concentration of plasma metabolites during an 8-h postprandial period. While postprandial differences in circulating metabolites can likely affect, e.g., satiety regulation, further studies are warranted to elucidate associations between the postprandial metabolome and endogenous responses.

## Figures and Tables

**Table 1:** Mean concentrations of plasma metabolites. q-values indicate false discovery rate (FDR)-adjusted *p*-values from mixed effect models for meal-time interactions (q_meal-time_), meal effect (q_meal_), and time effect (q_time_). Letters indicate significant differences in post-hoc tests at a given time point within each metabolite section.

Time (min)	0	30	60	90	120	180	240	300	360	420	480	q_meal-time_	q_meal_	q_time_
**Acetic acid**														
Cheese	0.070	0.063	0.036	0.029	0.030 ^ab^	0.025 ^a^	0.018 ^a^	0.030 ^a^	0.038 ^a^	0.036 ^a^	0.027 ^a^	<0.001	<0.001	<0.001
Hom. Cheese	0.057	0.046	0.029	0.017	0.023 ^a^	0.026 ^a^	0.026 ^a^	0.034 ^a^	0.032 ^a^	0.033 ^a^	0.034 ^ab^			
MCI Drink	0.057	0.044	0.024	0.027	0.033 ^ab^	0.023 ^a^	0.020 ^a^	0.028 ^a^	0.029 ^a^	0.031 ^a^	0.027 ^a^			
MCI Gel	0.066	0.050	0.037	0.032	0.050 ^b^	0.056 ^b^	0.073 ^b^	0.084 ^b^	0.095 ^b^	0.066 ^b^	0.057 ^b^			
**Acetoacetic acid**														
Cheese	0.070	0.063	0.036	0.029	0.030	0.025	0.018	0.030	0.038 ^ab^	0.036	0.027	<0.001	0.831	0.003
Hom. Cheese	0.057	0.046	0.029	0.017	0.023	0.026	0.026	0.034	0.032 ^ac^	0.033	0.034			
MCI Drink	0.057	0.044	0.024	0.027	0.033	0.023	0.020	0.028	0.029 ^c^	0.031	0.027			
MCI Gel	0.066	0.050	0.037	0.032	0.050	0.056	0.073	0.084	0.095 ^b^	0.066	0.057			
**Acetone**														
Cheese	0.035	0.035	0.033	0.037	0.036	0.039	0.036	0.031	0.032	0.035	0.036	0.112	0.992	0.001
Hom. Cheese	0.035	0.033	0.038	0.036	0.034	0.038	0.031	0.032	0.031	0.035	0.032			
MCI Drink	0.032	0.044	0.039	0.042	0.036	0.042	0.035	0.037	0.035	0.035	0.032			
MCI Gel	0.024	0.032	0.037	0.037	0.030	0.039	0.035	0.030	0.036	0.034	0.038			
**Citric acid**														
Cheese	0.148	0.157	0.156	0.151	0.142	0.139	0.153	0.148	0.145	0.147	0.162	0.061	0.992	<0.001
Hom. Cheese	0.143	0.157	0.167	0.161	0.126	0.150	0.141	0.139	0.139	0.162	0.150			
MCI Drink	0.142	0.161	0.149	0.141	0.149	0.137	0.156	0.141	0.141	0.153	0.158			
MCI Gel	0.130	0.166	0.157	0.148	0.127	0.150	0.148	0.158	0.148	0.165	0.168			
**Creatine**														
Cheese	0.008	0.013	0.011	0.010	0.007	0.007	0.005	0.005	0.005	0.005	0.005	0.963	0.992	<0.001
Hom. Cheese	0.007	0.014	0.010	0.012	0.005	0.009	0.007	0.008	0.008	0.006	0.006			
MCI Drink	0.010	0.014	0.012	0.011	0.006	0.006	0.007	0.005	0.005	0.006	0.003			
MCI Gel	0.007	0.013	0.012	0.011	0.009	0.008	0.008	0.005	0.007	0.006	0.006			
**Creatinine**														
Cheese	0.098	0.107	0.103	0.105	0.104	0.099	0.099	0.094	0.095	0.094	0.094	0.691	0.992	<0.001
Hom. Cheese	0.098	0.106	0.111	0.101	0.104	0.102	0.096	0.095	0.094	0.095	0.093			
MCI Drink	0.099	0.109	0.105	0.102	0.103	0.097	0.096	0.094	0.095	0.093	0.093			
MCI Gel	0.092	0.109	0.110	0.102	0.107	0.098	0.096	0.094	0.089	0.094	0.090			
**Dimethylsulfone**														
Cheese	0.008	0.007	0.009	0.010	0.007	0.008	0.008	0.007	0.007	0.007	0.007	0.649	0.992	0.043
Hom. Cheese	0.008	0.010	0.008	0.009	0.007	0.009	0.008	0.008	0.010	0.008	0.008			
MCI Drink	0.010	0.011	0.009	0.006	0.004	0.007	0.008	0.009	0.007	0.009	0.009			
MCI Gel	0.006	0.007	0.007	0.005	0.004	0.006	0.007	0.006	0.008	0.007	0.008			
**Formic acid**														
Cheese	0.020	0.021	0.024	0.024	0.015	0.019 ^ab^	0.023 ^a^	0.021	0.021 ^ab^	0.022 ^ab^	0.014 ^a^	0.212	0.041	0.061
Hom. Cheese	0.019	0.026	0.024	0.021	0.015	0.020 ^ab^	0.021 ^a^	0.022	0.019 ^ab^	0.021 ^a^	0.019 ^a^			
MCI Drink	0.022	0.022	0.019	0.026	0.018	0.016 ^a^	0.022 ^ab^	0.018	0.015 ^a^	0.020 ^a^	0.021 ^ab^			
MCI Gel	0.024	0.021	0.024	0.021	0.021	0.027 ^b^	0.030 ^b^	0.028	0.028 ^b^	0.030 ^b^	0.029 ^b^			
**Glucose**														
Cheese	5.533	6.514 ^a^	5.191	5.322	5.322 ^ab^	5.408	5.140	5.398	5.480	5.432	5.337 ^ab^	<0.001	0.992	<0.001
Hom. Cheese	5.554	6.338 ^ac^	5.270	5.103	5.123 ^a^	5.296	5.279	5.270	5.376	5.349	5.265 ^a^			
MCI Drink	5.808	5.500 ^b^	4.629	5.355	5.830 ^b^	5.564	5.411	5.518	5.428	5.462	5.465 ^b^			
MCI Gel	5.427	5.476 ^bc^	4.898	5.137	5.591 ^ab^	5.541	5.507	5.463	5.344	5.368	5.304 ^ab^			
**3-hydroxybutyric acid**														
Cheese	0.088	0.073	0.046	0.040	0.042	0.058	0.053	0.078	0.098 ^ab^	0.113	0.139	0.037	0.992	<0.001
Hom. Cheese	0.136	0.075	0.047	0.043	0.042	0.060	0.073	0.072	0.082 ^ab^	0.129	0.120			
MCI Drink	0.074	0.096	0.061	0.056	0.062	0.052	0.049	0.050	0.059 ^a^	0.085	0.084			
MCI Gel	0.074	0.094	0.059	0.042	0.049	0.063	0.080	0.079	0.121 ^b^	0.109	0.139			
**Lactic acid**														
Cheese	0.771	0.796	1.048 ^a^	1.067 ^a^	0.955	0.832	0.841	0.701	0.691	0.695	0.670	<0.001	0.438	<0.001
Hom. Cheese	0.807	0.828	1.100 ^a^	1.110 ^a^	1.023	0.850	0.797	0.685	0.678	0.717	0.724			
MCI Drink	0.835	0.737	0.807 ^b^	0.800 ^b^	0.884	0.826	0.854	0.720	0.690	0.634	0.643			
MCI Gel	0.778	0.740	0.831 ^b^	0.900 ^ab^	0.846	0.773	0.678	0.697	0.626	0.680	0.773			
**Pyruvic acid**														
Cheese	0.061	0.064	0.100 ^a^	0.103 ^a^	0.078	0.073	0.062	0.057	0.053	0.049	0.044	<0.001	0.992	<0.001
Hom. Cheese	0.062	0.068	0.091 ^ab^	0.103 ^a^	0.076	0.075	0.064	0.057	0.046	0.048	0.043			
MCI Drink	0.068	0.066	0.075 ^ab^	0.074 ^b^	0.073	0.076	0.073	0.065	0.054	0.045	0.045			
MCI Gel	0.064	0.056	0.071 ^b^	0.083 ^ab^	0.065	0.070	0.058	0.052	0.047	0.046	0.049			

## Data Availability

Pseudo-anonymized data can be made available upon request before 2029 via a data sharing contract. From 2029, fully anonymized data can be transferred.

## Supplementary Materials

The following are available online at https://www.mdpi.com/article/10.3390/nu13124280/s1, Table S1: Mean concentrations of plasma amino acids.
