# Supplementary material for: Effect of Dairy Matrix on the Postprandial Blood Metabolome
Source: Nutrients. 2021 Nov 27;13(12):4280. doi: 10.3390/nu13124280 (PMC8709269; doi:10.3390/nu13124280)
Supplement: Supplementary file 1 [file nutrients-13-04280-s001.zip › nutrients-1463791-supplementary.pdf]

Table S1. Mean concentrations of plasma amino acids. q-values indicate FDR-adjusted p-values from mixed effect models for meal-time interactions ($q_{meal\text{-}time}$), meal effect (q_{meal}) and time effect (q_{time}). * indicate $q < 0.05$. Letters indicate significant differences in post hoc tests at a given time point within each metabolite section.

Time (min)	0	30	60	90	120	180	240	300	360	420	480	$q_{meal\text{-}time}$	q_{meal}	q_{time}
**Alanine**														
Cheese	0.303	0.367	0.429	0.453	0.429	0.404	0.401	0.358	0.329	0.311	0.297	<0.001*	0.992	<0.001*
Hom. Cheese	0.322	0.386	0.439	0.470	0.454	0.437	0.398	0.354	0.333	0.314	0.296			
MCI Drink	0.324	0.419	0.446	0.413	0.399	0.395	0.393	0.373	0.359	0.321	0.305			
MCI Gel	0.319	0.388	0.426	0.428	0.394	0.375	0.358	0.330	0.293	0.283	0.290			
**Glutamine**														
Cheese	0.741	0.822	0.868	0.857	0.779	0.821	0.833	0.822	0.836	0.799	0.806	0.588	0.992	<0.001*
Hom. Cheese	0.733	0.821	0.886	0.870	0.780	0.850	0.816	0.802	0.816	0.809	0.781			
MCI Drink	0.724	0.876	0.873	0.834	0.703	0.819	0.831	0.838	0.826	0.822	0.814			
MCI Gel	0.679	0.829	0.883	0.840	0.734	0.823	0.826	0.825	0.807	0.794	0.791			
**Glycine**														
Cheese	0.251	0.272	0.275	0.264	0.265	0.247	0.238	0.221	0.227	0.217	0.221	0.328	0.992	<0.001*
Hom. Cheese	0.252	0.270	0.275	0.269	0.270	0.252	0.230	0.225	0.227	0.226	0.209			
MCI Drink	0.246	0.283	0.276	0.248	0.254	0.243	0.247	0.237	0.231	0.225	0.231			
MCI Gel	0.230	0.267	0.267	0.249	0.252	0.222	0.229	0.215	0.208	0.209	0.207			
**Histidine**														
Cheese	0.079	0.092	0.097	0.094	0.099	0.092	0.095	0.092	0.092	0.084	0.086	0.185	0.992	<0.001*
Hom. Cheese	0.072	0.088	0.101	0.099	0.097	0.097	0.094	0.088	0.086	0.089	0.079			
MCI Drink	0.076	0.099	0.100	0.092	0.108	0.089	0.088	0.087	0.085	0.082	0.080			
MCI Gel	0.072	0.093	0.098	0.097	0.095	0.092	0.092	0.088	0.088	0.083	0.076			
**Leucine**														
Cheese	0.109	0.164^{a}	0.199	0.208	0.191	0.184	0.177	0.158	0.158	0.143	0.143	0.001*	0.992	<0.001*
Hom. Cheese	0.108	0.155^{a}	0.188	0.203	0.205	0.204	0.190	0.161	0.146	0.133	0.133			
MCI Drink	0.099	0.207^{b}	0.212	0.199	0.193	0.180	0.158	0.152	0.145	0.132	0.131			
MCI Gel	0.096	0.192^{ab}	0.201	0.206	0.210	0.197	0.175	0.155	0.143	0.130	0.118			
**Isoleucine**														
Cheese	0.050	0.087^{a}	0.102^{a}	0.096^{a}	0.095	0.092	0.088	0.080	0.078	0.070	0.063	<0.001*	0.041*	<0.001*
Hom. Cheese	0.054	0.087^{a}	0.101^{a}	0.110^{ab}	0.116	0.104	0.095	0.088	0.067	0.059	0.056			

MCI Drink	0.054	0.126[b]	0.129[b]	0.118[b]	0.114	0.098	0.085	0.092	0.076	0.071	0.062			
MCI Gel	0.052	0.108[ab]	0.118[ab]	0.116[ab]	0.121	0.110	0.101	0.093	0.083	0.067	0.059			
**Methionine**														
Cheese	0.070	0.092[a]	0.106	0.111	0.080	0.097[ab]	0.094	0.087	0.078	0.069	0.074	0.588	0.041*	<0.001*
Hom. Cheese	0.068	0.092[a]	0.105	0.107	0.074	0.107[a]	0.100	0.089	0.084	0.072	0.067			
MCI Drink	0.066	0.117[b]	0.116	0.108	0.081	0.101[ab]	0.091	0.093	0.087	0.077	0.075			
MCI Gel	0.055	0.092[a]	0.109	0.100	0.068	0.090[b]	0.086	0.076	0.068	0.057	0.066			
**Phenylalanine**														
Cheese	0.035	0.052	0.057	0.060	0.062	0.055	0.051	0.043	0.042[a]	0.041[a]	0.034[a]	0.050	0.041*	<0.001*
Hom. Cheese	0.034	0.052	0.057	0.060	0.069	0.059	0.055	0.041	0.038[a]	0.032[ab]	0.023[ab]			
MCI Drink	0.037	0.061	0.056	0.059	0.058	0.052	0.049	0.043	0.035[ab]	0.031[ab]	0.026[ab]			
MCI Gel	0.029	0.053	0.056	0.055	0.054	0.054	0.047	0.040	0.020[b]	0.028[b]	0.017[b]			
**Tyrosine**														
Cheese	0.044	0.067[a]	0.081	0.090	0.089	0.086	0.083[ab]	0.077[ab]	0.072[a]	0.070[a]	0.058[a]	<0.001*	0.992	<0.001*
Hom. Cheese	0.044	0.066[a]	0.081	0.089	0.097	0.095	0.092[a]	0.079[a]	0.072[a]	0.063[ab]	0.056[a]			
MCI Drink	0.044	0.084[b]	0.086	0.092	0.083	0.083	0.077[b]	0.071[ab]	0.065[ab]	0.059[b]	0.054[ab]			
MCI Gel	0.044	0.076[ab]	0.089	0.091	0.090	0.088	0.078[ab]	0.068[b]	0.057[b]	0.055[b]	0.047[b]			
**Valine**														
Cheese	0.243	0.305[a]	0.344	0.370	0.351	0.355[a]	0.358	0.343	0.344	0.320	0.308	<0.001*	0.992	<0.001*
Hom. Cheese	0.259	0.315[a]	0.356	0.382	0.375	0.396[b]	0.376	0.360	0.345	0.318	0.304			
MCI Drink	0.251	0.366[b]	0.370	0.371	0.387	0.354[ab]	0.340	0.329	0.324	0.294	0.303			
MCI Gel	0.236	0.339[ab]	0.368	0.374	0.389	0.391[ab]	0.357	0.342	0.326	0.303	0.280			
**Total BCAA**														
Cheese	0.402	0.556[a]	0.645	0.674	0.638	0.630[a]	0.623	0.581	0.580	0.533	0.514	<0.001*	0.992	<0.001*
Hom. Cheese	0.422	0.557[a]	0.645	0.695	0.696	0.704[b]	0.661	0.609	0.558	0.509	0.493			
MCI Drink	0.404	0.700[b]	0.710	0.688	0.694	0.632[ab]	0.583	0.574	0.545	0.497	0.496			
MCI Gel	0.385	0.639[ab]	0.687	0.696	0.720	0.698[ab]	0.634	0.590	0.552	0.500	0.457			
